# Diversity and Functions of Endophytic Fungi Associated with Roots and Leaves of *Stipa purpurea* in an Alpine Steppe at Qinghai-Tibet Plateau

**DOI:** 10.4014/jmb.2002.02056

**Published:** 2020-04-23

**Authors:** Xiaoyan Yang, Hui Jin, Lihong Xu, Haiyan Cui, Aiyi Xin, Haoyue Liu, Bo Qin

**Affiliations:** 1 CAS Key Laboratory of Chemistry of Northwestern Plant Resources and Key Laboratory for Natural Medicine of Gansu Province, Lanzhou Institute of Chemical Physics, Chinese Academy of Sciences, Lanzhou 730000, P.R. China; 2 Qilihe District Agricultural Technology Extension Station of Lanzhou, Lanzhou 730000, P.R. China; 3 School of Forensic Medicine, Shanxi Medical University, Taiyuan 030001, P.R. China; 4 China Institute for Radiation Protection, Taiyuan 030006, P.R. China

**Keywords:** Endophytic fungi, *Stipa purpurea*, diversity, ACC deaminase activity, antimicrobial activity

## Abstract

*Stipa purpurea* is a unique and dominant herbaceous plant species in the alpine steppe and meadows on the Qinghai-Tibet Plateau (QTP). In this work, we analyzed the composition and diversity of the culturable endophytic fungi in *S. purpurea* according to morphological and molecular identification. Then, we investigated the bioactivities of these fungi against plant pathogenic fungi and 1- aminocyclopropane-1-carboxylate deaminase (ACCD) deaminase activities. A total of 323 fungal isolates were first isolated from *S. purpurea*, and 33 fungal taxa were identified by internal transcribed spacer primers and grouped into Ascomycota. The diversity of endophytic fungi in *S. purpurea* was significantly higher in roots as compared to leaves. In addition, more than 40% of the endophytic fungi carried the gene encoding for the ACCD gene. The antibiosis assay demonstrated that 29, 35, 28, 37 and 34 isolates (43.9, 53.1, 42.4, 56.1, and 51.5%) were antagonistic to five plant pathogenic fungi, respectively. Our study provided the first assessment of the diversity of culture- depending endophytic fungi of *S. purpurea*, demonstrated the potential application of ACCD activity and antifungal activities with potential benefits to the host plant, and contributed to high biomass production and adaptation of *S. purpurea* to an adverse environment.

## Introduction

With a mean altitude of over 4,000 m, the Qinghai-Tibet Plateau (QTP) is the highest plateau in the world and is sometimes called “the Third Pole of the Earth.” It covers approximately 2.5 million km2 and contains glaciers, alpine lakes and waterfalls [1]. There are harsh environmental conditions in the plateau habitat, such as strong wind, high ultraviolet radiation, poor soil nutrient availability, low temperature and precipitation. In such a harsh environment, the dominant vegetation types in the hinterland of the QTP are alpine steppe and alpine meadow, accounting for more than 60% of the area on the plateau [2]. Alpine steppes and meadows play a very important role in maintaining the living standard of people on the QTP.

*Stipa purpurea* is a unique and dominant herbaceous plant species in the alpine steppe and meadows on the QTP. It is rich in crude proteins and fats, which makes it an important natural forage resource of major ecological and economic value [3]. The plant has a strong ability to resist alpine environmental stresses, such as strong UV-B radiation, low temperature and drought [4]. As one part of the important alpine ecosystem, *S. purpurea* plays an important role in preserving and stabilizing diversity in the QTP.

Fungi that live in different organs (root, stem, leaf, flower, fruit, and seed) of a plant without causing visible damage to the host are known as endophytic fungi [5]. Some researchers have shown that the endophytic fungi communities are not only an important source of novel bioactive secondary metabolites, enzymes and biological control agents, but are also important sources of fungi with plant growth-promoting and antimicrobial activities [6-11]. Among these properties, the ability of microbes to produce 1-aminocyclopropane-1-carboxylate deaminase (ACCD) has been identified as one of the direct mechanisms of plant growth promotion. The ACCD- producing plant growth-promoting rhizobacteria (PGPR) not only protect the plant from the negative impact of high ethylene concentration under stress but may also enhance plant growth and development by a number of other mechanisms. These include facilitating nutrient uptake, production of phytohormones, and solubilization of nutrients. There is evidence for the beneficial effect of ACCD-producing PGPR in terms of stress (biotic and abiotic) amelioration, biofertilization, biocontrol, phytostimulation and bioremediation, all of which exert a positive impact on crop productivity and normal functioning of the ecosystem [12, 13]. Interestingly, it has been found that ACCD activity is not unique to bacteria. Evidence of ACCD activity was also detected in *Penicillium citrinum* and *Trichoderma* spp. [12, 14]. However, the role of ACCD in plant growth-promoting fungi (PGRF) has not been investigated in depth. In addition, many novel and valuable compounds with antimicrobial activity have been successfully obtained from endophytic fungi [15]. Studies have demonstrated that the endophytic fungi community lives in close relation with the plant host, influencing physiological processes and interaction with other organisms. Plant endophytes have been isolated from plants in flooded areas, arctic regions and deserts. It has also been noted that a given plant species can have at least a partially species-specific community of endophytic fungi [16].

At present, most studies of *S. purpurea* have focused on genetic diversity, community classification, physiological ecology and grassland community [1, 17-19]. Based on internal transcribed spacer rRNA cloning and sequencing methods, our previous study has demonstrated that a wide variety of fungal communities were associated with the roots and rhizosphere soil of *S. purpurea* and that the fungal assemblages are strongly influenced by different habitats [20]. Few studies have, however, focused on the diversity of endophytic fungal communities and their potential ecological functions. Therefore, we hypothesized that abundant endophytes associated with the roots and leaves of *S. purpurea* would have functions of PGRF, such as ACCD activity and antimicrobial activity. To test these hypotheses, we collected root and leaf samples of *S. purpurea* at multiple locations in the alpine steppe and meadows on the QTP, analyzed the composition and diversity of culture- dependent endophytic communities, and investigated the functional traits of endophytes with potential benefits to the host plant. The aims of this work are i) to characterize endophytic fungi communities found in *S. purpurea*; ii) to compare the fungal community diversity associated with specific tissues; iii) to determine the functional traits of endophytes with potential benefits to the host plant.

## Materials and Methods

### Collection of Samples and Isolation of Fungal Endophytes

*S. purpurea* plants were randomly collected in July 2014 from Gangcha steppe district in the Haibei Tibetan Autonomous Prefecture of Qinghai Province (37° 16'-37° 18'N, 100° 15'-100° 18'E, about 3,265 m a.s.l) where *S. purpurea* is the most dominant plant species. Identification of plants was simple since *S. purpurea* inflorescence was present at sampling time. Some basic properties of the sampling site and the detailed process of the sample collection were described in a previously published study [20]. All plant materials were immediately brought to the laboratory, stored at 4°C in a refrigerator and processed within 24 h.

Fresh and healthy green leaves were selected. Roots were selected if they were connected to green leaves, had root hairs, and did not have obvious lesions. The samples were rinsed under running water to remove soil and debris. More than 100 segments were randomly selected for fungal isolations. The segments were surface- sterilized with 75% ethanol for 30 sec, 2% NaOCl for 10 min to destroy the surface microbes and their DNA, rinsed three times with distilled water, and then cut into segments (5-8 mm). Aliquots of the final H2O rinse were inoculated on potato dextrose agar (PDA) media (Sigma-Aldrich, USA; containing : 200 g/l, dextrose 20 g/l, agar 20 g/l; pH 6.0), Sigma-Aldrich, USA, containing (g/l): potato 200, dextrose 20 and agar 20; pH 6.0) to verify that no surface microorganisms were present [21]. Around 6 segments of each plant tissue were plated on PDA media containing 50 ppm streptomycin sulfate and 100 ppm ampicillin sodium salt. These antibiotics were used to prevent bacterial growth. The plates were incubated in the dark for 5-7 days at 25°C. Emerging fungal colonies were recorded, transferred, and purified on PDA plates for 2 or 3 times [22]. All living isolates were preserved in a freezer at -18°C with conservative glycerine in the laboratory.

### Molecular Identification

Microbial DNA was extracted from fungal isolates by using the Plant DNA Extraction Kit (Tiangen Biotech, China) based on the method of Guo *et al*. [23]. The target regions of ITS rDNA were amplified by PCR (95°C for 3 min, followed by 35 cycles of 94°C for 30 sec, 52°C for 30 sec, and 72°C for 30 sec, and a final elongation at 72°C for 10 min) using the following primers: ITS1F (5’-CTT GGT CAT TTA GAG GAA GTA A-3’) and ITS4 (5’-TCC TCC GCT TAT TGA TAT GC-3’) [24]. PCR was performed in triplicate in mixtures containing 12.5 μl 2×MasterMix (Tiangen Biotech Co., Ltd, China), 0.5 μl of each primer (20 μM each), 0.5 μl template, and adding ddH2O to a final volume of 25 μl. Amplifications were performed using a Gene Amp PCR System 9700 (Applied Biosystems, USA). Positive and negative controls (no DNA template) were included in all experiments. The PCR products were tested in 1.2% (w/v) agarose gel. Then, the PCR products were purified using a Gel Product Purification Kit (Tiangen Biotech Co., Ltd.) and sequenced by Sangon Biotech Co., Ltd. (Shanghai, China). Fungal isolates were identified by comparison against the SILVA LSU database [25] and a BLASTn search of the nucleotide reference database (http://blast.ncbi.nlm.nih.gov/) [26]. Priority was given to sequences derived from authoritative materials, such as ex-type or ex-epitype cultures. The sequences of the present study were submitted to the NCBI GenBank database.

### Diversity Analysis

Colonization frequency (FC) (%) was determined as the ratio of the number of plant fragments colonized by fungi and the total number of fragments×100. Species richness and evenness were calculated by the endophyte diversity analysis. The species diversity was calculated by the methods of Shannon-Wiener, Simpson, and Chao1, while the Pearson product-moment correlation coefficient was used to analyze the possible correlations between the root and leaf endophytic fungi communities of *S. purpurea.* To test for the significant differences in the composition of fungal communities from plant tissue environments, we applied Kruskal–Wallis one-way analysis of variance (ANOVA) on ranks. Since the data were not normally distributed, nonparametric tests were used. All statistical analyses were done using Sigma Plot 12.5 (Systat Software, USA) and p values of less than 0.05 were considered to indicate statistical significance. The communities of the endophytic fungi in *S. purpurea* leaves and roots were related in the ordination of a correspondence analysis (USA). The data were assessed by SPSS 16 software (SPSS, USA).

### Functional Traits of Endophytes with Potential Benefits to the Host Plant

**PCR screening of genes for ACC deamination and evaluation of ACCD activity.** The *acd*S gene coding the ACCD was amplified from genomic DNA by specific PCR. The PCR-detection of *acd*S was PCR-amplified using the primers *acd*S3F (5´-ATC GGC GGC ATC CAG WSN AAY CAN AC-3´) and *acd*S3R (5´-GTG CAT CGA CTT GCC CTC RTA NAC NGG RT-3´). The PCR mixture had a final volume of 25 μl, as described above. Amplification conditions were: 4 min at 94°C, 35 cycles of 45 sec at 94°C, 45 sec at 52, 45 sec at 72°C and a final extension of 5 min at 72°C [27]. Amplified products were detected by 1.2% (w/v) agarose gel electrophoresis. According to the method of Viterbo *et al.* [12], ACCD activity was evaluated quantitatively by measuring the amount of α-ketobutyrate produced by the deamination of ACC. ACCD activity was expressed in μmol of α- ketobutyrate/mg-1 of protein/h-1. Protein concentrations were determined using the Bio-Rad (Promega) reagent. Three independent replicate flasks were analyzed. The experiment was repeated three times.

**Antagonistic assay in vitro against pathogenic fungi.** All the isolates of fungal endophyte were tested for antagonistic activity against five plant pathogenic fungi: *Alternaria alternata* [28], *Alternaria solani* [29], *Botrytis cinerea*, *Fusarium oxysporum* [30] and *Fusarium solani* [31] using the plate diffusion method [32]. All the plant pathogenic fungi were from the CAS Key Laboratory of Chemistry of Northwestern Plant Resources, Lanzhou Institute of Chemical Physics, Chinese Academy of Sciences [33]. The fungal isolates and fungal pathogens were co-cultured on a PDA plate. Briefly, 5 mm PDA block with 5-day-old mycelia of the pathogenic fungi was placed at the center of a 9 cm petri plate containing PDA and the isolates of fungal endophyte placed on the agar surface at 3 equidistant points, 2.5 cm from the periphery of the plate [32]. Dual cultures were incubated at 25°C for 5 days and the diameter of fungal mycelial growth was measured and compared to the control (with pathogenic fungi alone).

Each inhibition assay was performed in triplicates, and values were presented as the mean (± standard deviation). The growth inhibition rate was calculated by measuring the inhibition zone diameter, and the formula is as follows:


$${\text{Growth Inhibition rate (GI) (\%) = (F}}_{cd}{\text{-T}}_{cd}{\text{)/(F}}_{cd}{\text{-F}}_{o}\text{)}\times \text{100}$$

where F_cd_ is the fungal colony diameter on the control PDA plate, T_cd_ is the fungal colony diameter on the experimental PDA base plate, and Fo is the diameter of the test fungus agar disks (5 mm) . The growth inhibition of the five pathogenic fungi by the endophytic fungi was assessed by in SPSS 16 software (SPSS).

## Results

### Isolates, Sequence Data, Diversity and Abundance

A total of 323 fungal isolates were obtained from the different tissues of *S. purpurea*. These isolates were grouped into at least 33 fungal taxa (Table 1). The majority of ITS sequences from the fungal isolates did not show complete sequence identity with sequences present in GenBank, ranging from 0.2% to greater than 10% sequence variation. All the ITS sequences were deposited in GenBank, and the accession numbers are MK102637-MK102702 corresponding to individual isolates (Table S1).

Further classification by morphological and molecular techniques indicated the dominance of Ascomycota in *S. purpurea* root and leaf communities. The 33 taxa were classified into 8 orders and 5 classes, with the isolate numbers shown in Fig. S1. Hypocreales was by far the most frequent order, which comprised 47.89% of the isolate. Most Hypocreales corresponded to *Fusarium* and *Trichoderma*. The other fungal orders included Pleosporales, Chaetothyriales, Helotiales, Pezizales, Magnaporthales, Sordariales and Xylariales (Fig. S1A, 21B and Table S2).

**Fig. 1:**
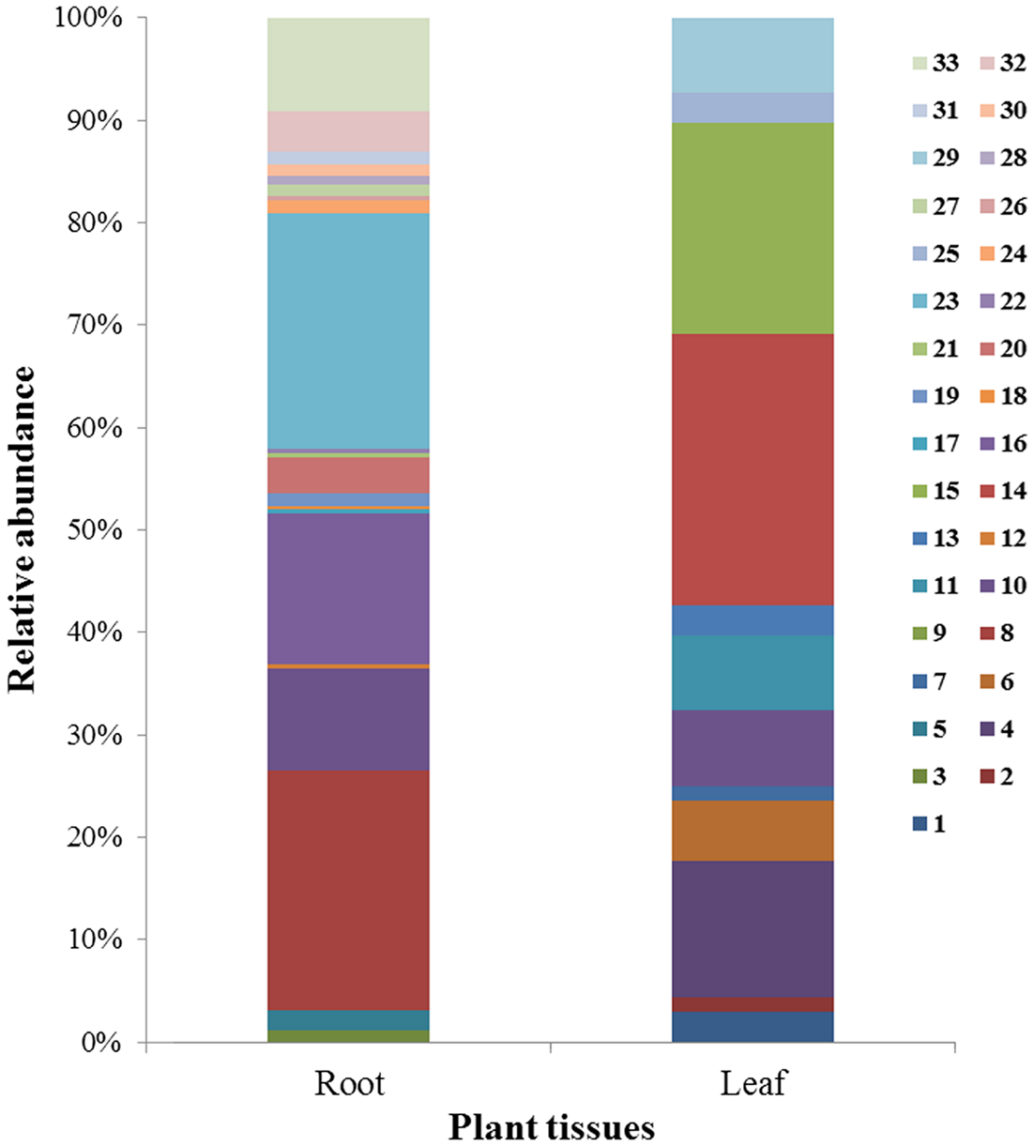
Relative abundance of fungal operational taxonomic units (OUTs) associated with *S. purpurea* with respect to the affiliation at the species level. 1, *Alternaria carthami*; 2, *Ascomycota* sp. ; 3, *Cadophora* sp.; 4, *Chaetomium murorum*; 5, *Darksidea delta*; 6, *Fusarium acuminatum*; 7, *Fusarium pseudograminearum*; 8, *Fusarium* sp.1; 9, *Fusarium* sp.2; 10, *Fusarium tricinctum*; 11, *Gloeotinia* sp.; 12, *Helotiales* sp.; 13, *Heydenia alpina*; 14, *Heydenia arietina*; 15, *Heydenia myrsines*; 16, *Microdochium bolleyi*; 17, *Monosporascus ibericus*; 18, *Myrothecium inundatum*; 19, *Paraphoma* sp.; 20, *Phialophora mustea*; 21, *Pleosporales* sp.; 22, *Pyricularia ctenantheicola*; 23, *Saccharicola bicolor*; 24, *Scytalidium* sp.; 25, *Simplicillium chinense*; 26, *Slopeiomyces cylindrosporus*; 27, *Stachybotrys bisbyi*; 28, *Stagonospora perfecta*; 29, *Thielavia hyalocarpa*; 30, *Trichoderma citrinum*; 31, *Trichoderm*a sp. ; 32, *Verticillium leptobactrum*; 33, *Verticillium* sp.

The most abundant 5 of 33 taxa were *Fusarium* sp. X, *Saccharicola bicolor*, *Microdochium bolleyi*, *Fusarium* sp. Y and *Verticillium* sp. These 5 taxa accounted for about 64% of the total isolate numbers (Fig. 1, Table S2). The isolates represented 5 classes at the class level. The class with the most abundant isolate numbers (IN) and strain types (ST) were Sordariomycetes (IN: 196, ST: 39), Dothideomycetes 71 (IN: 71, ST: 17), Pezizomycetes (IN: 34, ST: 4) Eurotiomycetes (IN: 9, ST: 1) and Leotiomycetes (IN: 12, ST: 5) (Figs. S1C and S1D, Table S2).

### The Fungal Community Diversity Associated with Specific Tissues

We found 252 isolates in root and 71 in leaf tissues (Tables 3 and S3). The root community consisted of at least 21 taxa. The leaf community was composed of 13 different taxa (Fig. 1). The diversity indices of these fungal endophytic communities showed relatively high diversity in richness, diversity, evenness, and species coverage (Table 3). Good’s coverage ranged from 97.1% to 97.6% of the total number of taxa. This indicated that most fungal ITS sequences detected were present in *S. purpurea* leaves. Root tissues had higher endophytic fungal species richness and diversity than leaf tissues (Table S3). Out of all the 33 taxa, only one taxon (*Fusarium* sp. Y) was isolated from both tissues. The most abundant taxa were *Fusarium* sp. in the roots and *Heydenia arietina* in the leaves.

The differences of species composition among plant tissue environments were examined using the Kruskal- Wallis ANOVA on ranks (Table S3). We tested the effect of plant tissue environments (total vs. root, total vs. leaf and root vs. leaf) on species composition. The results manifested that there was not a statistically significant difference of fungal endophytic species composition in root and as compared to leaf samples (*p* > 0.05). Furthermore, Pearson’s product-moment correlation coefficient indicated the species composition of total endophytic fungi had a positive relationship with the root samples (*p<* 0.0001), but no significant correlation to the leaf samples (*p* > 0.05). Endophytic fungi species composition did not show a significant correlation between the root and leaf samples (*p* > 0.05) (Table S3).

**Fig. 2:**
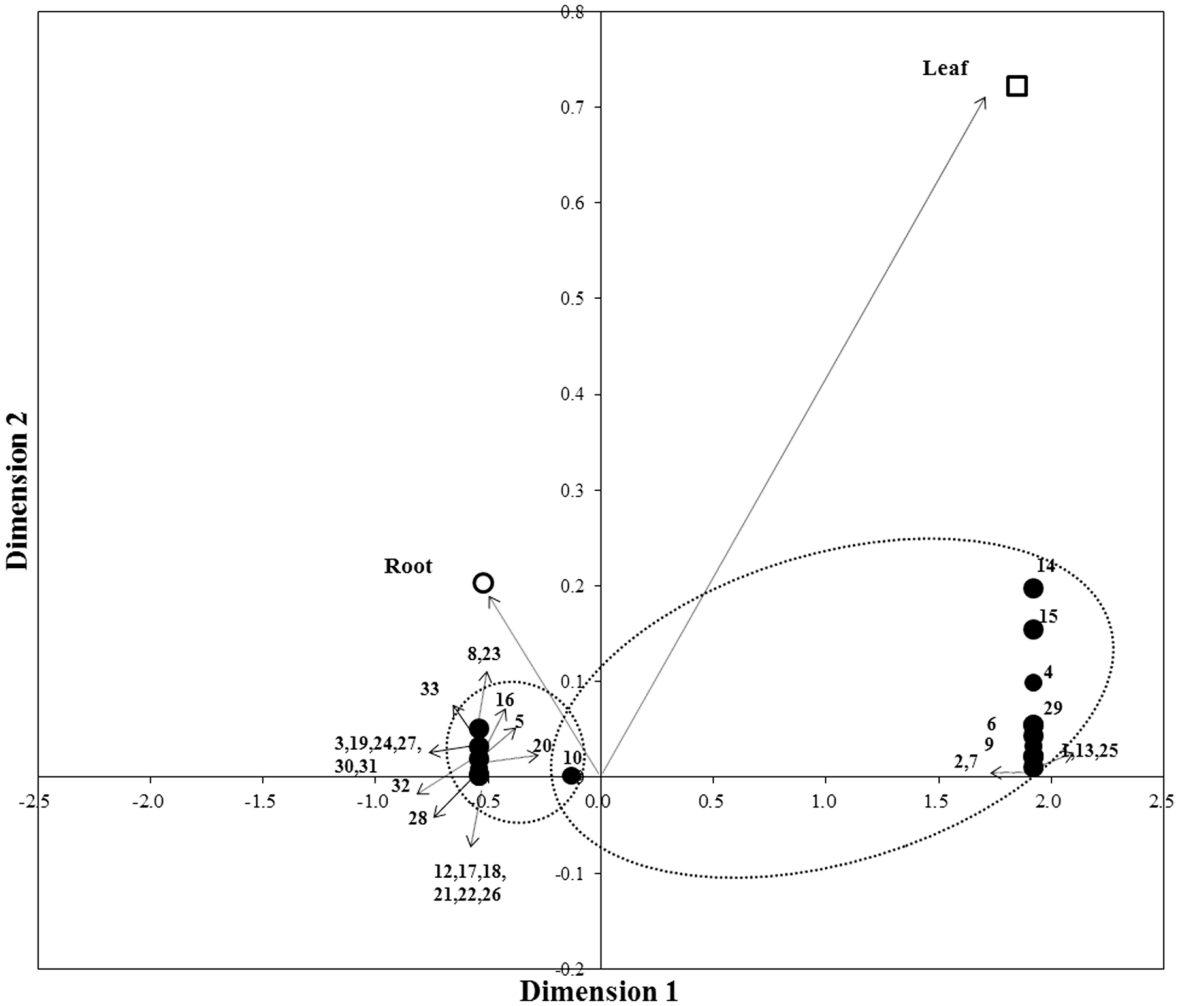
Correspondence analysis (CA) of the fungal community composition associated with *S. purpurea*. Two different clusters of samples are clearly shown in the CA diagram. Results for roots are clustered at the upper left (*x*, -0.521; *y*, 0.203) and results for leaves are at the upper right (*x*, 1.847; *y*, 0.722). Axes are correspondence components, DIM 1 and DIM2, with score and loading values. 1, *Alternaria carthami*; 2, *Ascomycota* sp. ; 3, *Cadophora* sp.; 4, *Chaetomium murorum*; 5, *Darksidea delta*; 6, *Fusarium acuminatum*; 7, *Fusarium pseudograminearum*; 8, *Fusarium* sp.1; 9, *Fusarium* sp.2; 10, *Fusarium tricinctum*; 11, *Gloeotinia* sp.; 12, *Helotiales* sp.; 13, *Heydenia alpina*; 14, *Heydenia arietina*; 15, *Heydenia myrsines*; 16, *Microdochium bolleyi*; 17, *Monosporascus ibericus*; 18, *Myrothecium inundatum*; 19, *Paraphoma* sp.; 20, *Phialophora mustea*; 21, *Pleosporales* sp.; 22, *Pyricularia ctenantheicola*; 23, *Saccharicola bicolor*; 24, *Scytalidium* sp.; 25, *Simplicillium chinense*; 26, *Slopeiomyces cylindrosporus*; 27, *Stachybotrys bisbyi*; 28, *Stagonospora perfecta*; 29, *Thielavia hyalocarpa*; 30, *Trichoderma citrinum*; 31, *Trichoderm*a sp. ; 32, *Verticillium leptobactrum*; 33, *Verticillium* sp.

CA was performed to analyze whether certain fungal species were related to a specific plant tissue environment (root or leaf). Differences in the fungal communities associated with the roots and leaves of *S. purpurea* were apparent (Fig. 2). Two cluster samples are clearly shown in the CA diagram. As shown by CA, the fungal community in the root of *S. purpurea* was correlated to sequences assigned to *Fusarium* sp.1, *S. bicolor*, *M. bolleyi*, *Fusarium tricinctum*, and *Verticillium sp.*; similarly, the fungal community in the leaf was correlated with sequences assigned to *H. arietina*, *Heydenia myrsines*, *Chaetomium murorum*, and *Thielavia hyalocarpa* (Fig. 2). CA also showed that for the 2 compartments, the structure of the fungal communities differed significantly by plant tissue environment: the diversity of the fungal community associated with roots was higher than that with the leaves of *S. purpurea.*

### Functional Traits of Endophytes with Potential Benefits to the Host Plant

As shown in Table 2, a positive PCR amplification of *acd*S was obtained for 15 isolates (42.86% of the endophytes of *S. purpurea*) belonging to *Paraphoma* (1), *Saccharicola* (1)*, Cadophora* (1), *Heydenia* (2), *Fusarium* (1), *Simplicillium* (1), *Trichoderma* (2), *Slopeiomyces* (1), *Chaetomium* (2), *Monosporascus* (1), and *Microdochium* (2) . ACCD activity of 15 isolates was determined. All of the 15 isolates exhibited different levels of ACCD activity, ranging from 0.15 to 7.22 μmol of α-ketobutyrate/mg-1 of protein/h-1. Among them, *Slopeiomyces cylindrosporus* ZMr08 showed the highest levels of ACCD activity, with 7.22 ± 2.6 μmol of α-ketobutyrate/mg-1 of protein/h-1.

The antagonistic activity of the endophytic fungi against the five pathogenic fungi was revealed by inhibition rate between 2.08 and 60.8%. *Fusarium pseudograminearum* ZMl17 was the strongest antagonist of *A. alternata* and *F. solani*, with inhibition zones of 45.8% and 60.8%, respectively. *Alternaria solani* was most strongly inhibited by the isolate *Trichoderma citrinum* ZMr18, which caused an inhibition zone of 55.5%. The isolate *Myrothecium inundatum* ZMr37 caused the largest inhibitory zone (56.3%) in *B. cinerea*, and *Monosporascus ibericus* ZMr17, the largest inhibition zone in *F. oxysporum* (50.0%).

Among the 33 species, 8 species had antagonistic effects on all the five plant pathogenic fungi. They are: *Paraphoma*, *S. bicolor*, *Fusarium* sp., *M. inundatum*, *Simplicillium chinense*, *T. citrinum*, *Verticillium* and *S. cylindrosporus*. *F. oxysporum* was the most susceptible fungal pathogen.

## Discussion

*S. purpurea*, as a dominant herbage resource in alpine steppe and meadows, has played an important role in the QTP [1, 3]. Research and application of endophytic fungi with growth-promoting activity are essential for the sustainable development of the alpine steppe and meadows [20]. To our best knowledge, there is currently no systematic and comprehensive study on the endophytic fungal community of *S. purpurea* and its related functions by culture-dependent methods.

One of the main aims of the study was to assess the diversity of the culturable endophytic fungal community of *S. purpurea*. Our research showed a wide range of endophytic fungi in *S. purpurea* leaves and roots (Table S2). Ascomycota dominated in these fungi. The results are similar to those of studies of the endophytic fungal communities of different plants by using culture-independent methods [34, 35]. In contrast, this is not consistent with previous reports of a large number of Basidiomycota endophytes in the root of *S. purpurea* by use of ITS cloning and sequencing [20]. This may be due to the limitation of the method used. Microorganisms are not all culturable [36].

In this study, the dominant endophytic fungal genera of *S. purpurea* were *Fusarium* and *Trichoderma*. These are very common soil fungi and are especially present in the rhizosphere and rhizoplane [37, 38]. *Fusarium* includes many plant, human, and insect pathogens as well as saprobes [39, 40] and had been investigated as a model of agriculturally relevant biological control fungi [41]. *Fusarium* species are common endophytes of *Phragmites australis* [42], *Ilex paraguariensis* [40], *Panax notoginseng* [43] and *Glycine max* [44]. *Trichoderma* contains important plant pathogen control agents and antimicrobial compound producers [45]. There are also some special fungal species in the root and leaf of *S. purpurea*, such as *Alternaria carthami*, *M. inundatum*, *Pyricularia ctenantheicola*, *S. cylindrosporus* and *Monosporascus ibericus*. Thus, *S. purpurea* hosts rich and diversified fungal resources [39]. There is an obvious difference between the occurrence frequencies of *S. cylindrosporus* with that previously reported [46]. This difference might be caused by environmental and abiotic stress. A large number of studies have shown that the environmental conditions of the alpine steppe and meadows in the QTP are relatively harsh [1, 16]. Therefore, specific isolates of *Fusarium*, *Trichoderma*, and some special rare taxa fungal species may be able to grow better under stressful conditions than other fungi, while establishing a more intimate relationship with *S. purpurea* [22, 38, 41].

Plant tissues are possible habitats for new fungal resources [5, 15]*.* Our study showed structural differences in the communities of culturable endophytic fungi colonizing *S. purpurea* leaves and roots with more fungal species in the roots than in the leaves. The dominant endophytic species in the different tissues of the plant also differed [47, 48]. *Fusarium* species are highly common endophytes that have been reported in almost all studied host plants. *Heydenia* species are in the leaf of *S. purpurea* (Rare taxa (RF<1%)) [47]*. Heydenia* species are found in extreme conditions, such as alpine habitats [49], lakes of maritime Antarctica [50], and soil and moss from the Taylor Valley in south Victoria Land of Antarctica [51]. This fact further confirmed the importance of studying the culturable endophytic fungi of *S. purpurea* under a harsh environment. Our study demonstrates that the endophytic fungi of *S. purpurea* are more abundant and diverse in roots than in leaves. This is consistent with previous research results [34, 42]. Some studies showed that the roots of perennial plants would have more time to accumulate endophytes from the soil and rhizosphere [52]. The prevalence and diversity of soil-borne fungi is higher than those infecting the aerial parts of the plant [17, 42, 48]. As *S. purpurea* is an excellent pasture plant, overgrazing has brought enormous pressure on the *S. purpurea* steppe. Since the root was the main place for endophytic fungi to survive, this might be correlated with its presence as the dominant herbage resource in alpine steppe and meadows on the QTP (Table 1).

Data from this study and previous reports revealed that the diversity of the endophytic fungal community in the root of *S. purpurea* was significant when internal transcribed spacer rRNA cloning and sequencing approaches were compared to culture approaches. The endophyte communities show only a 7.5% overlap in the dominant phyla or species between these two approaches [20]. The culture-dependent approaches are heavily biased towards fast-growing species, and many fungi have specialized growth requirements, so this approach may be biased in favor of the dominant species [36]. The ITS rRNA cloning library approach to a wide range of substrates has resulted in the identification of a large number of new fungal species that cannot be cultivated, many of which constitute novel lineages or are dominant components of microbial communities. Both approaches show that the host is a strong diversifier of fungal community structure, but that each alone covers only a small proportion of the real fungal community [53].

The knowledge of the culturable endophytic community composition is of great value for the development of technologies for agricultural management, in particular those related to microorganism-based growth promotion, since the isolates are available for activity and inoculation analyses [40]. In this study, the potential of endophytic isolates for plant growth promotion was determined by ACCD gene detection and fungal antagonism. Given the importance of *S. purpurea* for the alpine steppe and meadows ecosystem, these results will contribute to knowledge for maximizing growth and health of forage. The isolates with ACCD activity are of much concern as PGPF. This enzyme makes use of the ethylene precursor, leading to a reduction in the ethylene concentration of plants and a consequent decrease in its negative effect on plant growth. Thus, the plants can better resist the stress of adverse environmental conditions such as drought, heavy metal, saline-alkali, and the presence of phytopathogens [54]. However, it should be emphasized that the research on ACCD-containing PGPR mainly focuses on the plants that are ethylene sensitive, such as canola, peppers and tomatoes [13, 55]. Therefore, it is necessary to identify sources of ACCD, such as in various adverse and extreme environmental conditions. *S. purpurea* is the dominant species in alpine arid and semi-arid meadows because of its outstanding tolerance to harsh environments (including cold, drought, and wind). Transcriptome analysis reveals a diversified adaptation of *S. purpurea* along a drought gradient on the QTP [1]. In our study, almost 40% of the isolates had the potential to produce ACCD. Species of these genera were also are considered as potential fungal inoculants for ACCD activities [12, 14]. In previous studies, it was demonstrated that the utilization ability of ACC was the most effective mechanism involved in root elongation and endophytic colonization by the isolates [12, 40]. Therefore, further study on ACCD-producing endophytic fungi of *S. purpurea* might also effectively reveal their adaptive mechanism under special circumstances and promote the growth of this forage.

In our study, a fairly high percentage (63%) of the 66 fungal isolates had obvious antagonistic properties, suggesting that the endophytic fungi of *S. purpurea* can resist certain microbial diseases. An increasing number of findings have indicated that new natural products from native grass-associated fungi exhibit antifungal activity [56]. Isolates from *Fusarium*, *Trichoderma*, *Verticillium*, *Myrothecium* and *Simplicillium* were found to have high antifungal activities. Although *Fusarium* as endophytes are normally in latent form but readily attack plants when conditions allow, they are a source of agricultural antibiotics and a biological control agent [7, 38, 41]. Members of the genus *Trichoderma* and *Verticillium* are reported as prolific sources for the production of natural products and many possess antifungal activity. So far, *Trichoderma* spp. are among the most studied fungal-based of biocontrol agents (BCAs) and are commercially marketed as biopesticides, biofertilizers and soil amendments [57]. *Simplicillium* species, as bio-control agents of phytopathogenic fungi and plant parasitic nematodes, have demonstrated positive effects [58]. These have been isolated mainly from soil [59]. As the alpine steppe and meadows are rarely affected by application of pesticides, this natural habitat contains richness in microbial resources [60]. Therefore, the endophytic fungi isolated from these especially can be one of the sources of plant disease biological control agents. This result is in agreement with previous reports on plants with strong resistance to fungal infection which have become an important source of antagonistic fungi [61].

What makes *S. purpurea* so successful even under apparently harsh environmental conditions has been the subject of much speculation. Our current findings on the cultivable endophytic fungi community composition and diversity and the beneficial traits microbes possess have furnished some evidence on the potential role of these microbes on the forage biomass production and the adaption of *S. purpurea* to adverse environments. The dual activities of microbial inhibition and plant growth promotion found in the endophytic isolates of *Fusarium*, *Trichoderma*, *Verticillium* and *Simplicillium* may provide *S. purpurea* with its adaptation to the adverse environment in the alpine steppe of the QTP and lay the foundation for the development of natural germicidal products.

## Supplemental Materials



Supplementary data for this paper are available on-line only at http://jmb.or.kr.

## Figures and Tables

**Table 1:** List of identified isolates, their abundances, origin and the frequency of colonization (FC%).

Species name	Numbers of isolates and their origin		FC (%)

	Root	Leaf		
*Alternaria carthami*	0	2	0.62
*Darksidea delta*	5	0	1.55
*Paraphoma* sp.	3	0	0.93
*Pleosporales* sp.	1	0	0.31
*Saccharicola bicolor*	58	0	17.96
*Stagonospora perfecta*	2	0	0.62
*Phialophora mustea*	9	0	2.79
*Cadophora* sp.	3	0	0.93
*Gloeotinia* sp.	0	5	1.55
*Helotiales* sp.	1	0	0.31
*Scytalidium* sp.	3	0	0.93
*Heydenia alpina*	0	2	0.62
*Heydenia arietina*	0	18	5.57
*Heydenia myrsines*	0	14	4.33
*Fusarium acuminatum*	0	4	1.24
*Fusarium pseudograminearum*	0	1	0.31
*Fusarium tricinctum*	25	5	9.29
*Fusarium* sp.1	59	0	18.27
*Fusarium* sp.2	0	3	0.93
*Myrothecium inundatum*	1	0	0.31
*Stachybotrys bisbyi*	3	0	0.93
*Simplicillium chinense*	0	2	0.62
*Trichoderma citrinum*	3	0	0.93
*Trichoderma* sp.	3	0	0.93
*Verticillium leptobactrum*	10	0	3.10
*Verticillium* sp.	23	0	7.12
*Pyricularia ctenantheicola*	1	0	0.31
*Slopeiomyces cylindrosporus*	1	0	0.31
*Chaetomium murorum*	0	9	2.79
*Monosporascus ibericus*	1	0	0.31
*Thielavia hyalocarpa*	0	5	1.55
*Microdochium bolleyi*	37	0	11.46
*Ascomycota* sp.	0	1	0.31

**Table 2:** Antifungal activity against five plant pathogenic fungi and ACCD activity of the fungal endophytes from *Stipa purpurea.*

Fungal growth inhibition^*^ (%)							ACCD activity^**^	

Isolates	Identified as	*Botrytis cinerea*	*Alternaria alternata*	*Alternaria solani*	*Fusarium oxysporum*	*Fusarium solani*	ACCD (*acd*S)	ACCD activity (μmol/mg per hour α-ketobutyrate)
ZMl09	*Alternaria carthami*	0	0	0	6.25±5.5	34.38±9.1	-	
ZMr04	*Paraphoma* sp.	54.1±5.1	38.9±6.4	45.7±7.9	28.6±7.4	38.2±11.8	+	0.23±0.05
ZMr11	*Paraphoma* sp.	33.3±6.6	34.7±5.7	32.9±7.0	0	0	-	
ZMr23	*Pleosporales* sp.	0	0	0	9.4±5.8	18.8±6.4	-	
ZMr02	*Saccharicola bicolor*	6.2±1.8	40.3±7.5	34.3±7.0	26.2±6.5	54.9±14.0	-	
ZMr12	*Saccharicola bicolor*	5.9±0.2	43.2±5.8	33.5±8.3	25.3±10.4	53.2±14.8	+	0.43±0.08
ZMr20	*Saccharicola bicolor*	6.4±0.3	39.8±6.0	32.5±7.3	23.5±8.3	52.2±13.9	-	
ZMr21	*Saccharicola bicolor*	6.1±0.1	42.2±6.2	35.7±10.0	29.9±7.9	56.7±14.3	-	
ZMr24	*Saccharicola bicolor*	5.9±0.1	42.5±7.3	32.1±7.8	28.7±8.0	58.8±15.7	-	
ZMr29	*Saccharicola bicolor*	5.8±0.1	44.6±6.6	33.6±8.7	23.3±7.9	55.9±15.0	-	
ZMr35	*Saccharicola bicolor*	6.3±0.2	38.9±6.7	37.8±7.1	24.4±7.9	51.2±14.2	-	
ZMr38	*Saccharicola bicolor*	5.7±0.1	42.3±7.0	36.7±7.2	23.5±8.7	53.2±13.8	-	
ZMr48	*Saccharicola bicolor*	6.4±0.3	43.4±19.8	35.7±7.9	25.6±9.5	56.7±14.4	-	
ZMr30	*Stagonospora perfecta*	0	20.8±7.5	28.6±6.8	16.7±7.5	42.2±11.1	-	
ZMr44	*Stagonospora perfecta*	0	21.3±6.1	29.8±9.5	15.9±7.9	43.2±11.2	-	
ZMr28	*Phialophora mustea*	2.1±0.9	18.1±6.7	28.6±9.3	9.5±6.4	0	-	
ZMr40	*Cadophora* sp.	25.0±1.7	36.1±8.3	45.7±7.1	26.2±8.3	0	+	0.27±0.06
ZMr33	*Helotiales* sp.	0	37.5±7.5	44.3±7.2	16.7±7.9	52.0±14.7	-	
ZMl04	*Heydenia alpina*	0	30.6±7.5	38.6±8.7	21.4±8.7	0	+	0.32±0.11
ZMl14	*Heydenia alpina*	0	32.3±6.9	40.9±7.7	20.1±9.1	0	-	
ZMl15	*Heydenia arietina*	0	0.0	42.9±7.9	28.6±7.9	0	+	0.29±0.09
ZMl13	*Heydenia myrsines*	0	34.7±7.9	34.3±7.3	19.0±7.4	0	-	
ZMl07	*Fusarium acuminatum*	30.0±6.6	0	0	9.4±7.5	28.1±8.6	-	
ZMl17	*Fusarium pseudograminearum*	0	45.8±7.9	51.4±8.1	28.6±9.1	60.8±15.6	-	
ZMl10	*Fusarium tricinctum*	35.42±6.9	0	32.9±7.2	0	55.9±14.7	-	
ZMl11	*Fusarium tricinctum*	36.6±6.	0	30.3±7.9	0	57.7±16.0	-	
ZMl12	*Fusarium tricinctum*	0	0	33.2±7.5	0	52.2±13.6	-	
ZMl16	*Fusarium tricinctum*	40.0±7.6	0	35.5±8.3	0	59.5±14.8	-	
ZMr01	*Fusarium tricinctum*	52.0±9.1	0	38.6±8.3	11.9±7.9	54.9±13.8	-	
ZMr14	*Fusarium* sp.	20.8±6.2	0	28.6±8.3	9.5±7.8	0	-	
ZMr47	*Fusarium* sp.	0	41.7±8.0	48.6±8.1	28.6±7.2	54.9±14.9	-	
ZMl03	*Fusarium* sp.	41.7±7.0	40.3±8.3	40.0±8.6	4.8±5.9	50.0±13.3	+	1.59±0.39
ZMr37	*Myrothecium inundatum*	56.3±6.2	37.5±6.9	44.3±7.2	16.7±7.5	58.8±16.2	-	
ZMl06	*Simplicillium chinense*	25.0±6.2	25.0±3.3	44.3±8.7	11.9±8.3	57.8±15.2	+	2.03±0.58
ZMr39	*Trichoderma citrinum*	0	40.3±7.5	54.3±9.7	19.0±8.3	58.8±16.2	-	
ZMr18	*Trichoderma citrinum*	0	41.2±5.4	55.5±8.3	18.1±8.7	57.6±14.5	+	0.63±0.15
ZMr19	*Trichoderma* sp.	0	0	0	0	0	+	0.59±0.19
ZMr25	*Verticillium leptobactrum*	0	0	0	6.3±6.2	35.9±10.1	-	
ZMr26	*Verticillium* sp.	0	4.5±5.2	0	34.4±8.9	42.2±11.5	-	
ZMr08	*Slopeiomyces cylindrosporus*	16.6±5.6	40.3±7.8	41.4±8.1	19.0±9.9	56.9±14.3	+	7.22±2.6
ZMl18	*Chaetomium murorum*	0	0	0.0	14.3±7.1	59.8±15.7	+	1.77±0.65
ZMl08	*Chaetomium murorum*	0	0	0.0	13.2±7.9	60.6±16.1	+	1.63±0.39
ZMr17	*Monosporascus ibericus*	0	27.8±7.9	35.7±7.6	50.0±7.8	32.4±8.4	+	0.29±0.09
ZMr03	*Microdochium bolleyi*	0	0	0	0	0	+	0.15±0.06
ZMr16	*Microdochium bolleyi*	0	0	0	0	0	+	0.19±0.08

* Growth inhibition 96 h after culture against five plant pathogenic fungi in dual cultures, Results are mean ± SD of three determinations.

** Detection of gene encoding for acdS enzyme ACCD (1-aminocyclopropane-1-carboxylate deaminase), ACCD activity was measured in μmol of α-ketobutyrate per milligram of protein per hour. The results are the mean of three replicates.

**Table 3:** Comparison of fungal taxa diversity in root and leaf samples by using of Chao 1 richness estimate, Shannon diversity index, Simpson index, Evenness and Coverage.

Sample	No. of numbers	No. of fungal taxa	Chao 1	Shannon	Simpson	Evenness	Coverage (%)
Root	252	21	29.25	2.219	0.858	0.86	97.63
Leaf	71	13	13.31	2.208	0.848	0.73	97.14

The Chao 1, Shannon’s diversity, Simpson diversity index, Shannon’s evenness and Good’s coverage richness estimators were calculated using the species data.
