# Supplementary material for: Diversity and Functions of Endophytic Fungi Associated with Roots and Leaves of Stipa purpurea in an Alpine Steppe at Qinghai-Tibet Plateau
Source: J Microbiol Biotechnol. 2020 Apr 23;30(7):1027–36. doi: 10.4014/jmb.2002.02056 (PMC9728210; doi:10.4014/jmb.2002.02056)
Supplement: Supplementary file 1 [file JMB-30-7-1027-supple.pdf]

J. Microbiol. Biotechnol.
https://doi.org/10.4014/jmb.2002.02056

Table S1

Taxon designation of fungal endophytes from roots and leaves of *S. purpurea* based on sequence data from the internal transcribed spacer regions of nuclear ribosomal RNA (ITS rRNA).

Genus (stated in GenBank)	Phylum; Subclass; Order;	Isolates	ITS (No.)*	Most closely related species**	% similarity
Darksidea	Ascomycota; Dothideomycetes; Pleosporales	ZMr06	MK102695	*Darksidea delta* (KP183980)	97.23
Saccharicola	Ascomycota; Dothideomycetes; Pleosporales	ZMr02	MK102684	*Saccharicola bicolor* (KT367526)	100.00
Saccharicola	Ascomycota; Dothideomycetes; Pleosporales	ZMr12	MK102685	*Saccharicola bicolor* (AF455415)	100.00
Saccharicola	Ascomycota; Dothideomycetes; Pleosporales	ZMr21	MK102687	*Saccharicola bicolor* (KP117300)	100.00
Saccharicola	Ascomycota; Dothideomycetes; Pleosporales	ZMr24	MK102686	*Saccharicola bicolor* (KP276515)	100.00
Saccharicola	Ascomycota; Dothideomycetes; Pleosporales	ZMr29	MK102688	*Saccharicola bicolor* (MH087100)	100.00
Saccharicola	Ascomycota; Dothideomycetes; Pleosporales	ZMr35	MK102689	*Saccharicola bicolor* (KM979977)	100.00
Saccharicola	Ascomycota; Dothideomycetes; Pleosporales	ZMr38	MK102690	*Saccharicola bicolor* (JX981466)	99.35
Saccharicola	Ascomycota; Dothideomycetes; Pleosporales	ZMr48	MK102691	*Saccharicola bicolor* (KJ188730)	99.58
Stagonospora	Ascomycota; Dothideomycetes; Pleosporales	ZMr30	MK102693	*Stagonospora perfecta* (KF251258)	98.48
Stagonospora	Ascomycota; Dothideomycetes; Pleosporales	ZMr44	MK102694	*Stagonospora perfecta* (KF251258)	97.23
Paraphoma	Ascomycota; Dothideomycetes; Pleosporales	ZMr04	MK102697	*Paraphoma* sp. (KT269100)	99.56
Paraphoma	Ascomycota; Dothideomycetes; Pleosporales	ZMr11	MK102698	*Paraphoma* sp. (KT269033)	100.00
Undefined genus	Ascomycota; Dothideomycetes; Pleosporales	ZMr23	MK102696	*Pleosporales* sp. (KT269765)	99.56
Phialophora	Ascomycota; Eurotiomycetes; Chaetothyriales	ZMr28	MK102700	*Phialophora mustea* (JX145398)	99.81
Cadophora	Ascomycota; Leotiomycetes; Helotiales	ZMr07	MK102699	*Cadophora* sp. (KT264475)	99.23
Cadophora	Ascomycota; Leotiomycetes; Helotiales	ZMr40	MK102701	*Cadophora* sp. (KT268419)	98.89
Scytalidium	Ascomycota; Leotiomycetes; Helotiales	ZMr13	MK102670	*Scytalidium* sp. YG-2010a (HQ213805)	99.35
Undefined genus	Ascomycota; Leotiomycetes; Helotiales	ZMr33	MK102702	*Helotiales* sp. r339 (HQ649857)	99.84

Trichoderma	Ascomycota; Sordariomycetes; Hypocreales	ZMr18	MK102662	*Trichoderma citrinum* (DQ865099)	92.45
Trichoderma	Ascomycota; Sordariomycetes; Hypocreales	ZMr39	MK102663	*Trichoderma citrinum* (KF887057)	99.39
Trichoderma	Ascomycota; Sordariomycetes; Hypocreales	ZMr19	MK102667	*Trichoderma* sp. (KP714590)	95.89
Verticillium	Ascomycota; Sordariomycetes; Hypocreales	ZMr25	MK102671	*Verticillium leptobactrum* (KJ188658)	98.56
Verticillium	Ascomycota; Sordariomycetes; Hypocreales	ZMr26	MK102672	*Verticillium* sp. TF17TTW (FJ948142)	97.49
Fusarium	Ascomycota; Sordariomycetes; Hypocreales	ZMr01	MK102655	*Fusarium tricinctum* (JQ846085)	97.56
Fusarium	Ascomycota; Sordariomycetes; Hypocreales	ZMr27	MK102656	*Fusarium tricinctum* (KF010839)	99.21
Fusarium	Ascomycota; Sordariomycetes; Hypocreales	ZMr09	MK102659	*Fusarium* sp. (JQ388254)	100.00
Fusarium	Ascomycota; Sordariomycetes; Hypocreales	ZMr41	MK102657	*Fusarium* sp. (JQ388252)	99.13
Fusarium	Ascomycota; Sordariomycetes; Hypocreales	ZMr42	MK102660	*Fusarium* sp. (AY924269)	99.78
Fusarium	Ascomycota; Sordariomycetes; Hypocreales	ZMr47	MK102661	*Fusarium* sp. (HQ130669)	99.45
Fusarium	Ascomycota; Sordariomycetes; Hypocreales	ZMr05	MK102658	*Fusarium* sp. (KT270287)	99.49
Myrothecium	Ascomycota; Sordariomycetes; Hypocreales	ZMr37	MK102665	*Myrothecium inundatum* (JQ936267)	99.59
Stachybotrys	Ascomycota; Sordariomycetes; Hypocreales	ZMr22	MK102664	*Stachybotrys bisbyi* (KP256004)	99.53
Stachybotrys	Ascomycota; Sordariomycetes; Hypocreales	ZMr14	MK102666	*Stachybotrys* sp. HZ-23 (EU301653)	99.54
Pyricularia	Ascomycota; Sordariomycetes; Magnaporthales	ZMr10	MK102669	*Pyricularia ctenantheicola* (KM484878)	99.79
Slopeiomyces	Ascomycota; Sordariomycetes; Magnaporthales	ZMr08	MK102668	*Slopeiomyces cylindrosporu* (JF508361)	98.46
Monosporascus	Ascomycota; Sordariomycetes; Sordariales	ZMr17	MK102683	*Monosporascus ibericus* (JQ973832)	99.47
Microdochium	Ascomycota; Sordariomycetes; Xylariales	ZMr03	MK102681	*Microdochium bolleyi*(KT692593)	98.12
Microdochium	Ascomycota; Sordariomycetes; Xylariales	ZMr15	MK102679	*Microdochium bolleyi* (KC989068)	96.41
Microdochium	Ascomycota; Sordariomycetes; Xylariales	ZMr16	MK102676	*Microdochium bolleyi* (JQ658340)	99.45
Microdochium	Ascomycota; Sordariomycetes; Xylariales	ZMr31	MK102678	*Microdochium bolleyi* (AM502264)	99.78
Microdochium	Ascomycota; Sordariomycetes; Xylariales	ZMr32	MK102682	*Microdochium bolleyi* (KT692593)	99.56
Microdochium	Ascomycota; Sordariomycetes; Xylariales	ZMr34	MK102677	*Microdochium bolleyi* (MH858255)	99.37
Microdochium	Ascomycota; Sordariomycetes; Xylariales	ZMr36	MK102673	*Microdochium bolle*yi (KP859010)	99.69
Microdochium	Ascomycota; Sordariomycetes; Xylariales	ZMr43	MK102674	*Microdochium bolleyi* (KJ188678)	99.41

Microdochium	Ascomycota; Sordariomycetes; Xylariales	ZMr45	MK102680	*Microdochium bolleyi* (HQ703412)	100.00
Microdochium	Ascomycota; Sordariomycetes; Xylariales	ZMr46	MK102675	*Microdochium bolleyi* (KF646098)	99.32
Alternaria	Ascomycota; Dothideomycetes; Pleosporales	ZMl09	MK102650	*Alternaria carthami* (KJ704213)	99.47
Gloeotinia	Ascomycota; Leotiomycetes; Helotiales	ZMl01	MK102637	*Gloeotinia* sp. LJC-2011(JF461264)	97.48
Heydenia	Ascomycota; Pezizomycetes ;Pezizales	ZMl04	MK102651	*Heydenia alpina* (JX171178)	95.25
Heydenia	Ascomycota; Pezizomycetes; Pezizales	ZMl13	MK102653	*Heydenia myrsines* (KU325128)	97.14
Heydenia	Ascomycota; Pezizomycetes; Pezizales	ZMl14	MK102654	*Heydenia alpina* (KU325118)	97.86
Heydenia	Ascomycota; Pezizomycetes; Pezizales	ZMl15	MK102652	*Heydenia arietina* (KF574887)	99.25
Simplicillium	Ascomycota; Sordariomycetes; Hypocreales	ZMl06	MK102638	*Simplicillium chinense* (KP034998)	97.47
Fusarium	Ascomycota; Sordariomycetes; Hypocreales	ZMl03	MK102645	*Fusarium* sp. MPG-5 (KC894839)	99.56
Fusarium	Ascomycota; Sordariomycetes; Hypocreales	ZMl07	MK102641	*Fusarium acuminatum* (KT070871)	99.47
Fusarium	Ascomycota; Sordariomycetes; Hypocreales	ZMl17	MK102640	*Fusarium pseudograminearum* (KP726905)	100.00
Fusarium	Ascomycota; Sordariomycetes; Hypocreales	ZMl10	MK102642	*Fusarium tricinctum* (JX406512)	99.47
Fusarium	Ascomycota; Sordariomycetes; Hypocreales	ZMl11	MK102643	*Fusarium tricinctum* (KU350729)	99.18
Fusarium	Ascomycota; Sordariomycetes; Hypocreales	ZMl12	MK102644	*Fusarium tricinctum* (KJ598865)	99.19
Fusarium	Ascomycota; Sordariomycetes; Hypocreales	ZMl16	MK102639	*Fusarium tricinctum* (KJ598867)	95.15
Chaetomium	Ascomycota; Sordariomycetes; Sordariales	ZMl08	MK102648	*Chaetomium murorum* (JQ946413)	98.89
Chaetomium	Ascomycota; Sordariomycetes; Sordariales	ZMl18	MK102649	*Chaetomium murorum* (KT192199)	97.48
Thielavia	Ascomycota; Sordariomycetes; Sordariales	ZMl05	MK102647	*Thielavia hyalocarpa* (JQ781752)	97.59
Undefined genus	Ascomycota;	ZMl02	MK102646	*Ascomycota* sp. (KP698348)	96.19

[*]ITS rDNA sequences of cultural endophytic fungi were deposited at GenBank;

[**]Matches of ITS rDNA sequences published were also from GenBank.

Table S2

The distribution of fungal taxa and isolate number at species, order, and class level.

Class type (5) and isolate number	Order type (8) and isolate number	OTUs(33) at species level and isolate number
**Dothideomycetes(71)**	Pleosporales（71）	*Alternaria carthami* (2), *Darksidea delta* (5), *Paraphoma* sp.(3), *Pleosporales* sp. (1), *Saccharicola bicolor* (58), *Stagonospora perfecta* (2),
**Eurotiomycetes(9)**	Chaetothyriales(9)	*Phialophora mustea* (9),
**Leotiomycetes(12)**	Helotiales(12)	*Cadophora* sp.(3), *Gloeotinia* sp. (5), *Helotiales* sp. (1), *Scytalidium* sp. (3),
**Pezizomycetes(34)**	Pezizales(34)	*Heydenia alpina*(2), *Heydenia myrsinea*(14), *Heydenia arietina*(18),
**Sordariomycetes(196)**	Hypocreales(142)	*Fusarium acuminatum*(4), *Fusarium pseudograminearum*(1), *Fusarium* sp.(62), *Fusarium tricinctum*(30), *Myrothecium inundatum*(1), *Simplicillium chinense*(2), *Stachybotrys bisbyi*(3), *Trichoderma citrinum*(3), *Trichoderma* sp. (3), *Verticillium leptobactrum*(10), *Verticillium* sp.(23) ,
	Magnaporthales(2)	*Pyricularia ctenantheicola*(1), *Slopeiomyces cylindrosporus*(1),
	Sordariales(15)	*Chaetomium murorum*(9), *Monosporascus ibericus*(1), *Thielavia hyalocarpa*(5), *Microdochium bolleyi*(37),
	Xylariales(37)	Undefined genus,
***Ascomycota* sp.(1)**		

Table S3

The effects of Kruskal-Wallis one-way ANOVA on ranks and Pearson product moment correlation on the endophytic fungal communities of *S. purpurea*.

Samples*	Kruskal-Wallis one-way ANOVA on ranks		Pearson Product Moment Correlation	
	H	*p*	CC**	*p*
Total vs. R	0.564	0.453	**0.967**	**<0.0001**
Total vs. L	0.441	0.507	0.283	0.461
R vs. L	0.002	0.964	0.029	0.941

Values in italics indicate significant differences or significant correlations

* R, root; L, leaf

**CC, correlation coefficient

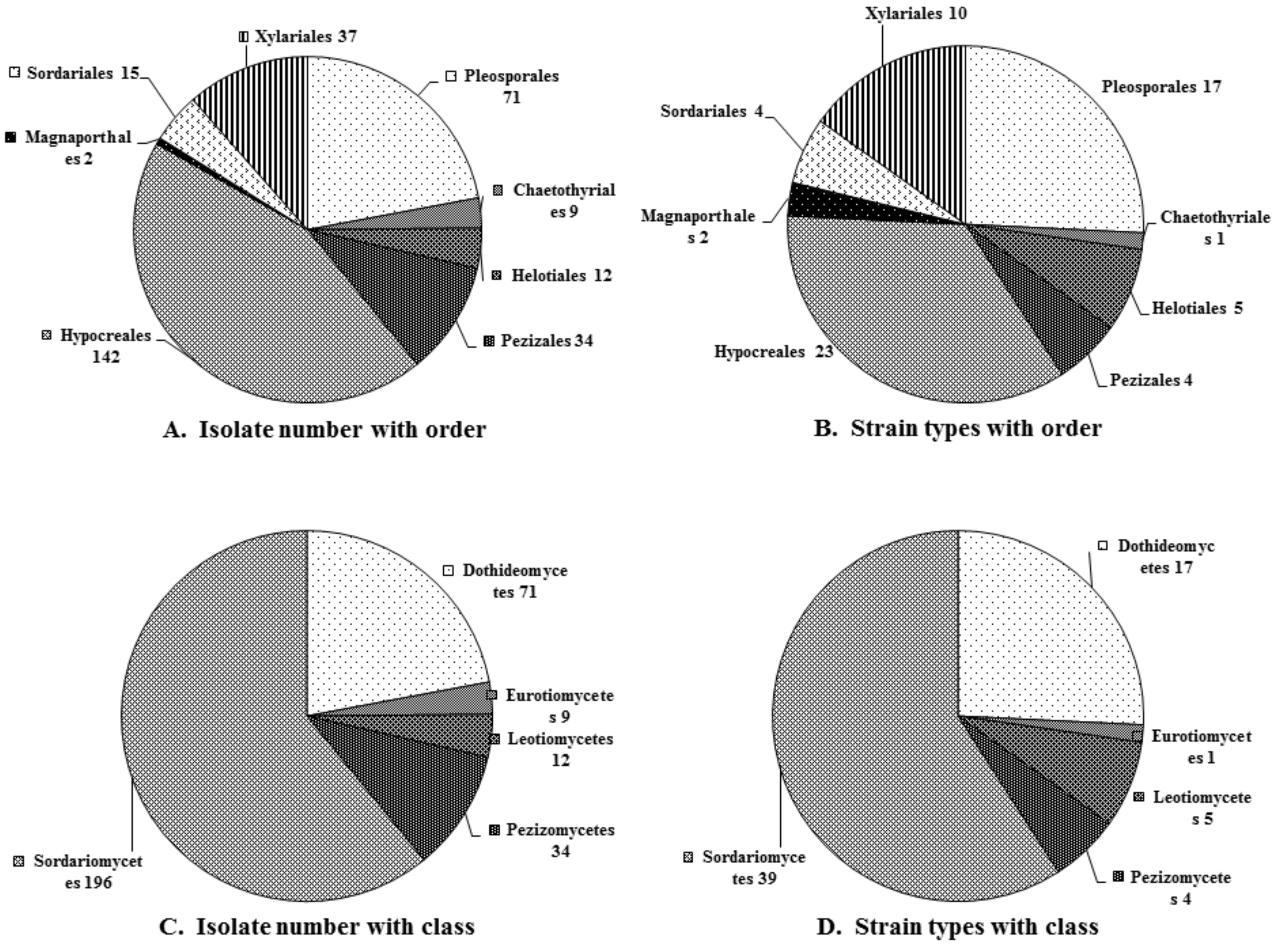

Fig. S1 The distribution of endophytic fungal isolates from *S. purpurea*. The isolate numbers (A) and strain types (B) at the order level; and the isolate numbers (C) and strain types (D) at the class level.
